# Supplementary material for: Mannan-Based Nanodiagnostic Agents for Targeting Sentinel Lymph Nodes and Tumors
Source: Molecules. 2020 Dec 31;26(1):146. doi: 10.3390/molecules26010146 (PMC7795445; doi:10.3390/molecules26010146)
Supplement: Supplementary file 1 [file molecules-26-00146-s001.pdf]

Supplementary Materials

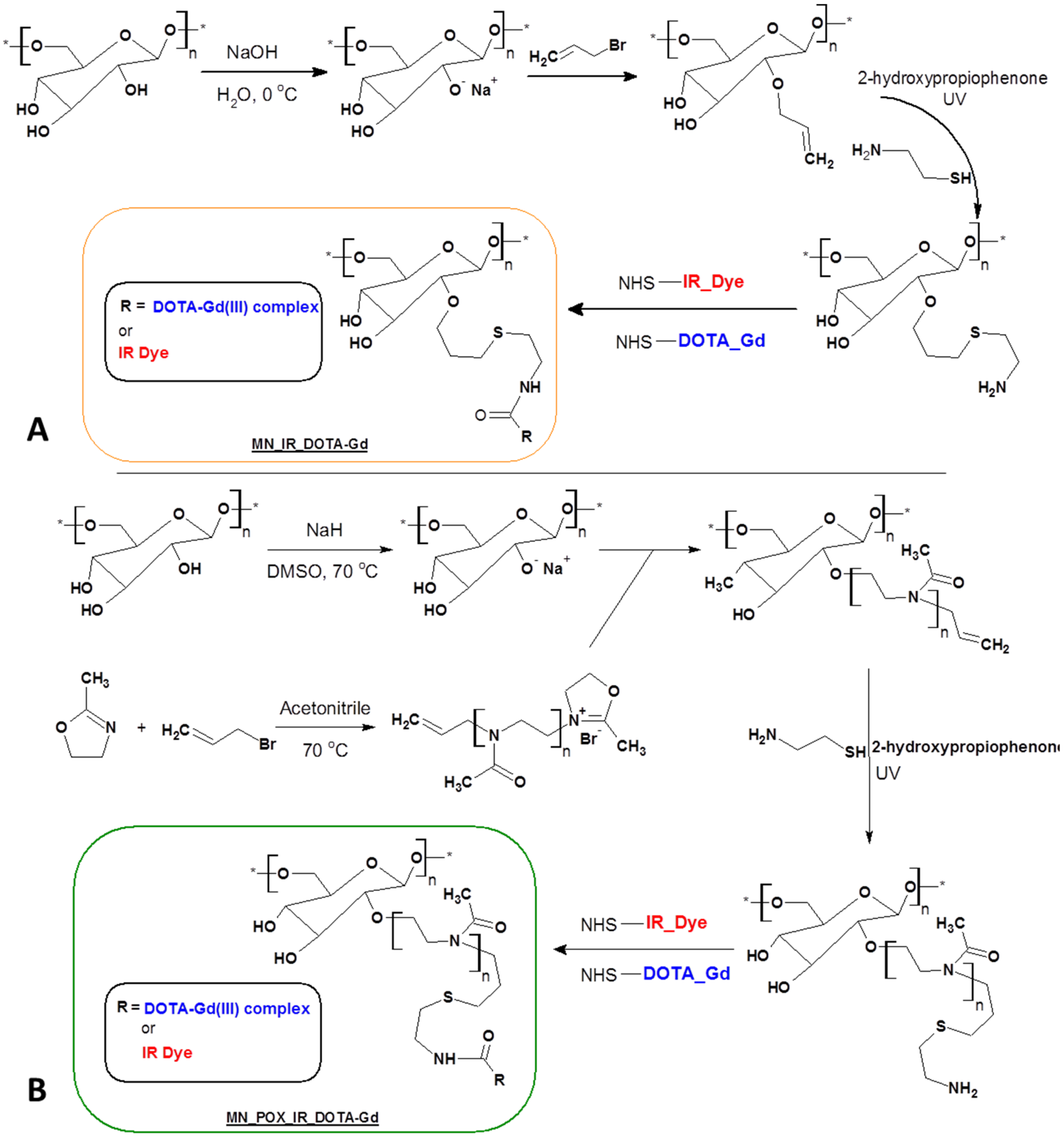

Figure S1. Synthesis paths for mannan modification. Preparation of (A) MN_IR_DOTA-Gd (means MN-DOTAGd-IR800) and (B) MN_POX_IR_DOTA-Gd (means MN-pMeOx-DOTAGd-IR800). [REF 38] reproduced by permission of The Royal Society of Chemistry (https://pubs.rsc.org/en/content/articlelanding/2018/tb/c7tb02888a#!divAbstract).

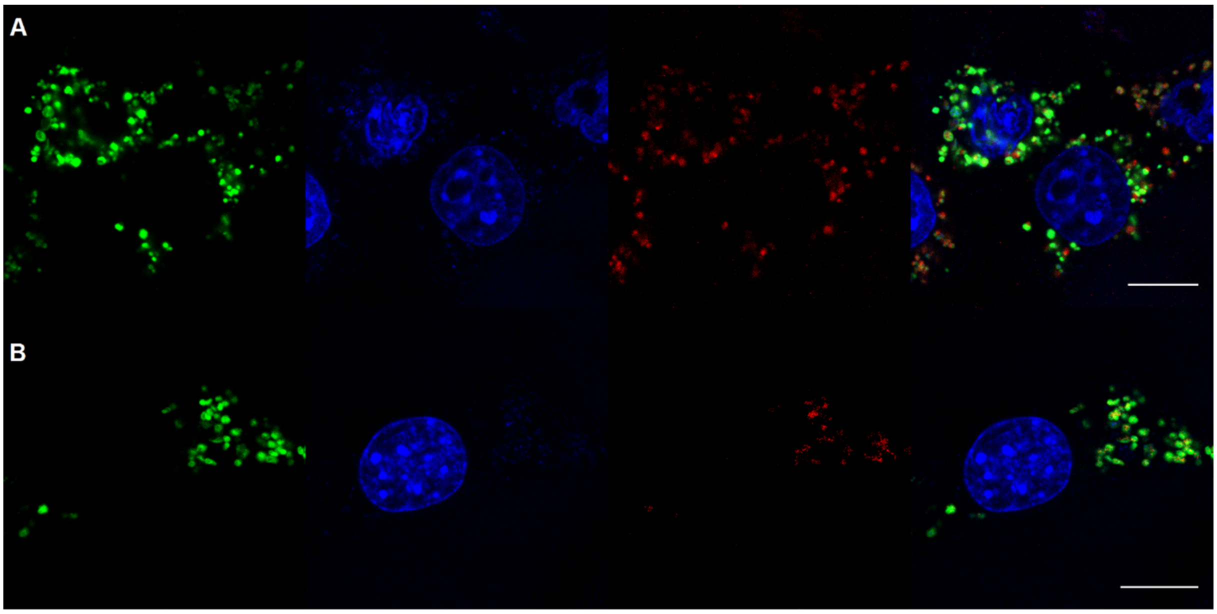

Figure S2. Representative confocal microscopy images: (A) 4T1 cells incubated with MN-DOTA Gd-IR800CW (MN), LysoTracker® Green and Hoechst 33342; (B) 4T1 cells incubated with MN-PMeOx-DOTA Gd-IR800CW (MNOX), LysoTracker® Green and Hoechst 33342. From left to right: LysoTracker® Green (green signal), Hoechst 33342 (blue signal), MN or MNOX (red signal originating from the fluorescent dye IR800CW), merged signals from all channels. Scale bar represents 10 μm.

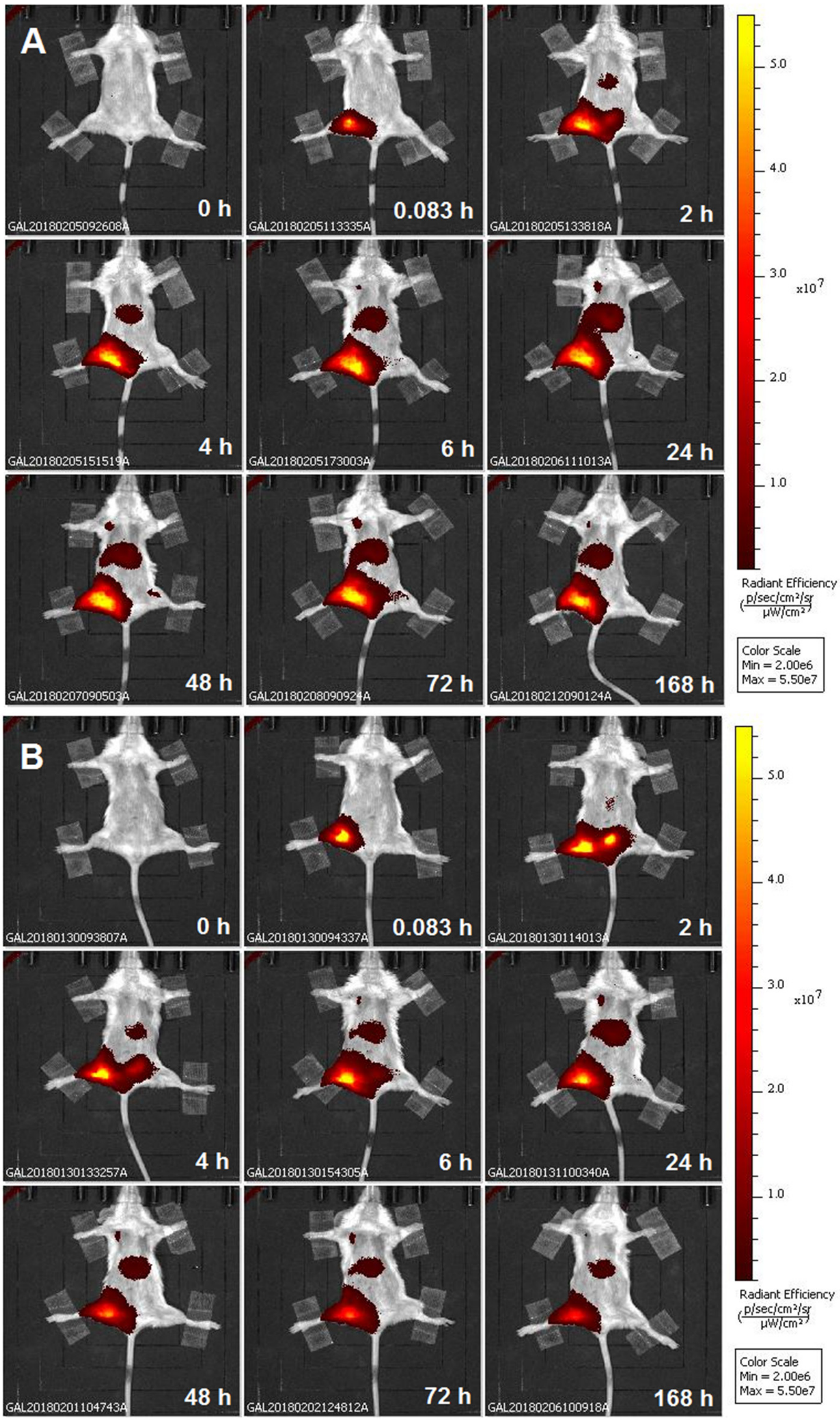

Figure S3. Change in fluorescence signal in vivo in time (representative example). 0 h point represents animal before MN or MNOX application. (A) Representative example of MN in mouse. (B) Representative example of MNOX in mouse.

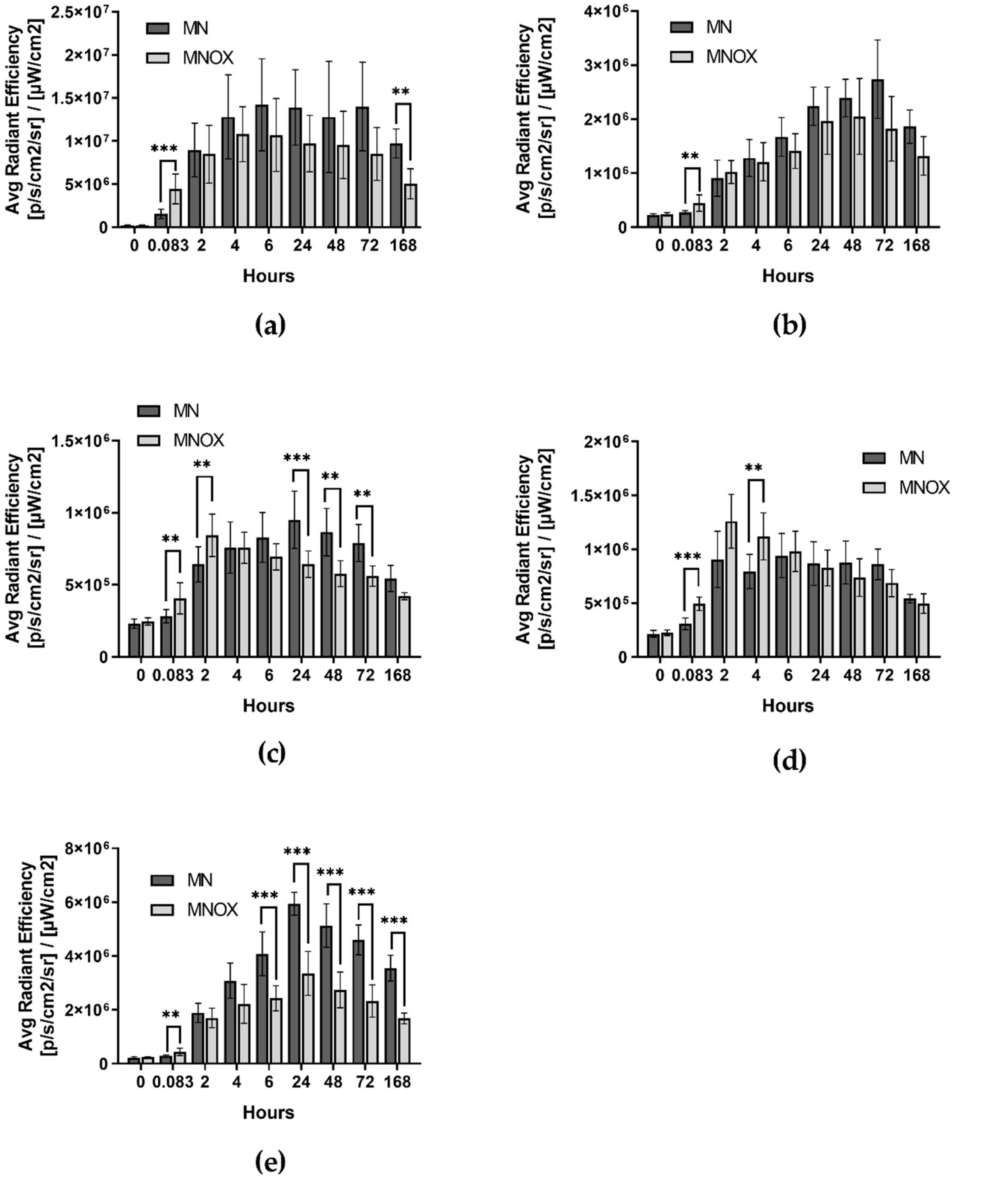

Figure S4. Quantification of in vivo fluorescence signals (all time points). Fluorescence signals was measured in vivo from (a) SLNs, (b) axillary lymph nodes on tumor sites, (c) axillary lymph nodes on non-tumor sites, (d) inguinal lymph nodes on non-tumor sites and (e) livers. The fluorescent signal is represented as the average radiant efficiency (mean + SD), p-values: *** $p<0.001$, ** $p<0.01$, * $p<0.05$.

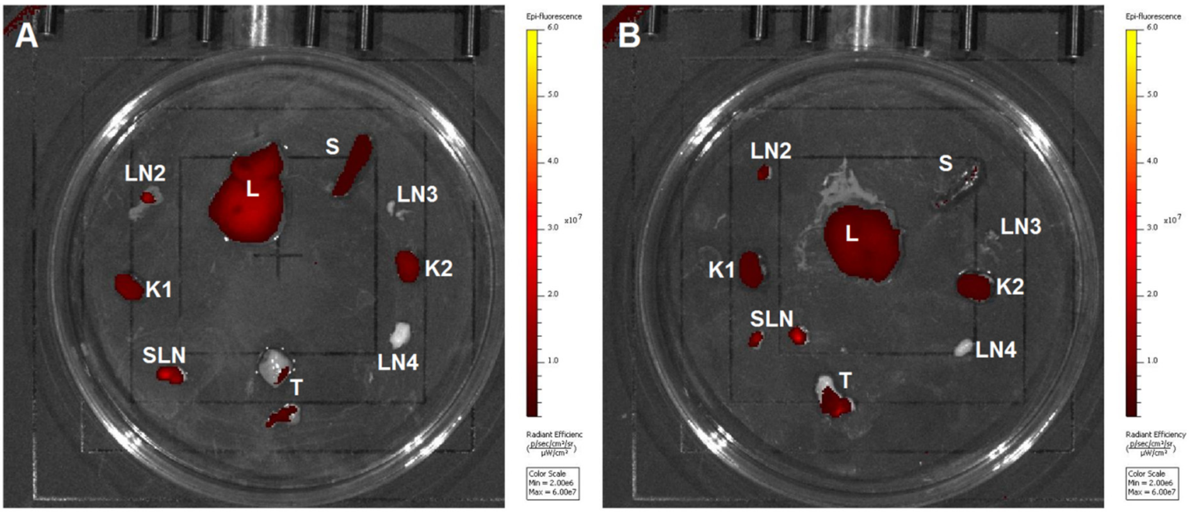

Figure S5. Representative example of ex vivo fluorescence signal on day 3 (after the application of MN/MNOX).(A) MN, (B) MNOX. L livers, SLN sentinel lymph node, LN2 axillary lymph node on tumor site, LN3 axillary lymph node on non-tumor site, LN4 inguinal lymph node on non-tumor site, S spleen, K1 kidney on tumor site, K2 kidney on non-tumor site,T tumor.
